# 3D Structure Modeling of Alpha-Amino Acid Ester Hydrolase from Xanthomonas rubrilineans

**Published:** 2013

**Authors:** S.A. Zarubina, I.V. Uporov, E.A. Fedorchuk, V.V. Fedorchuk, A.V. Sklyarenko, S.V. Yarotsky, V.I. Tishkov

**Affiliations:** Department of Chemical Enzymology, Faculty of Chemistry, M.V. Lomonosov Moscow State University; Leninskie gory, 1/3, Moscow, Russian Federation, 119991; Innovations and High Technologies MSU Ltd, Tsimlyanskya Str., 16, office 96, Moscow, Russian Federation, 109559; A.N. Bach Institute of Biochemistry, Russian Academy of Sciences, Leninskiy prospect, 33/2, Moscow, Russian Federation, 119071; State Research Institute for Genetics and Selection of Industrial Microorganisms (GosNIIgenetika), 1-st Dorozhniy pr., 1, Moscow, Russian Federation, 117545

**Keywords:** alpha-amino acid ester hydrolase, Xanthomonas, rubrilineans, computer simulation, docking, enzymatic synthesis of antibiotics, protein engineering

## Abstract

Alpha-amino acid ester hydrolase (EC 3.1.1.43, AEH) is a promising biocatalyst for the production of semi-synthetic β-lactam antibiotics, penicillins and cephalosporins. The AEH gene from *Xanthomonas rubrilineans* (XrAEH) was recently cloned in this laboratory. The three-dimensional structure of XrAEH was simulated using the homology modeling method for rational design experiments. The analysis of the active site was performed, and its structure was specified. The key amino acid residues in the active site - the catalytic triad (Ser175, His341 and Asp308), oxyanion hole (Tyr83 and Tyr176), and carboxylate cluster (carboxylate groups of Asp209, Glu310 and Asp311) - were identified. It was shown that the optimal configuration of residues in the active site occurs with a negative net charge -1 in the carboxylate cluster. Docking of different substrates in the AEH active site was carried out, which allowed us to obtain structures of XrAEH complexes with the ampicillin, amoxicillin, cephalexin, *D*-phenylglycine, and 4-hydroxy-*D*-phenylglycine methyl ester. Modeling of XrAEH enzyme complexes with various substrates was used to show the structures for whose synthesis this enzyme will show the highest efficiency.

## INTRODUCTION

Semi-synthetic β-lactam antibiotics are widely used to treat pathogens and make up more than half of the world market of antibacterial drugs [1]. These antibiotics are currently produced using the penicillin acylase (PA) enzyme, which catalyzes the reaction of acyl group transfer from the corresponding amide to the β-lactam nucleus (*Scheme*) [2, 3]. In the case of PA, the role of acyl moiety donors is played by amides, which are less reactive than the corresponding ethers. Therefore, the formation of an acyl-enzyme (stage with constant *k2*) can proceed much faster when the corresponding ester is used as a source of the acyl group, but this requires using a hydrolase instead of an amidase, such as PA. Hydrolase is more active with ethers, amide being the target product. Hence, the rate of the hydrolytic side reaction (stage with constant *k5*) catalyzed by hydrolase is lower compared to that of hydrolysis by amidase. This should increase the ratio between the synthesis and hydrolysis reaction rates. Thus, the use of hydrolase instead of amidase improves the efficiency of antibiotics synthesis in both steps.

**Scheme 1:**
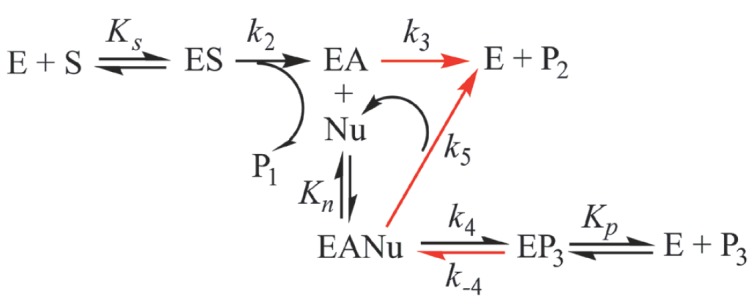
The common kinetic scheme of β-lactam antibiotic synthesis [2]. E – enzyme; S – substrate, donor of acyl moiety; ES – enzyme-substrate complex; EA – acylenzyme; P1 and P2 – products of substrate S hydrolysis; Nu – nucleophile; EANu – complex of acyl-enzyme with nucleophile; EP3 – complex of enzyme with target antibiotic; P3 – target antibiotic. *KS *– dissociation constant of the enzyme-substrate complex; *Kn *– dissociation constant of complex of acyl-enzyme with nucleophile; *Kp *– dissociation constant of enzyme with antibiotic synthesis product;* k2 *– rate constant of acyl-enzyme formation; *k3 *– rate constant of acyl-enzyme hydrolysis; *k4*, *k−4 *– forward and reverse rate constants of the chemical formation stage and target antibiotic hydrolysis, respectively; *k5 *– hydrolysis rate constant of the complex of acyl-enzyme with nucleophile

One such is hydrolase specific to α-amino acids esters (AEH, [EC 3.1.1.43]). Penicillin acylases have been isolated from various sources and well characterized; however, the data on AEH are scarce. Some data is available on AEH isolated from bacteria *Acetobacter turbidans *ATCC 9325 (ActAEH) [4, 5] and *Xanthomonas citri *IF0 3835 (XcAEH) [6], *X. campestris pv. campestris* ATCC 33913 [7], and a number of other sources. The Protein Data Bank (PDB) contains three-dimensional structures of only two enzymes that exhibit the highest activity in antibiotics synthesis - ActAEH and XcAEH. Only a structure of holo-form is available for XcAEH (PDB ID: 1MPX, resolution 1.9 A) [6]. In the case of ActAEH, there are structures of both, holo-form of wild type enzyme (PDB ID: 2B9V, resolution 2.0 A), and its complex with D-phenylglycine (PDB ID: 2B4K, 3.3 A), as well as structures of the mutant ActAEH Y206A (PDB ID: 1RYY, resolution 2.8 A) and complex of an inactive mutant ActAEH S205A with ampicillin (PDB ID: 1NX9, resolution 2.2 A) [5].

We recently cloned the AEH gene from bacteria *X. rubrilineans *(XrAEH). This strain was discovered at the State Scientific Center for Antibiotics. The enzyme has been successfully expressed in *Escherichia coli *cells; preliminary experiments have confirmed the high efficacy of recombinant XrAEH in the synthesis of several antibiotics. However, additional experiments on XrAEH engineering are required to ensure efficient practical use of the enzyme. The experiments should be focused on improving the enzyme’s properties with specified substrates. The rational design method is one of the most efficient approaches in protein engineering. This method involves introducing point amino acid substitutions into a protein globule, which are selected according to data obtained by analyzing the enzyme 3D structure. This method requires the availability of the structure of the enzyme under study, which can be obtained either experimentally (XRD or NMR) or through a computer simulation. The latter approach is now being used increasingly frequently thanks to the development of computer simulation methods and the continuous increase in the number of experimentally determined structures in the PDB data bank.

The purpose of this study was to build a model structure of XrAEH of holo-form of enzyme as well as complexes with the key compounds used for the synthesis of β-lactam antibiotics.

## EXPERIMENTAL

The amino acid sequences of XrAEH and known AEH structures were aligned using the BioEdit Sequence Alignment Editor ClustalW Multiple Alignment program [8].

A computer model of the three-dimensional structure of XrAEH was obtained with the homology modeling method using the Insight II software package. The structure of AEH from *X. citri *(XcAEH), available in the PDB database, code 1MPX (resolution of 1.9 A) [6], was used as a reference structure. The structure was further optimized using the molecular mechanics method (Discover_3 module of the Insight II software package, 300 steps of minimization, CVFF force field [9]) to relieve the potential conformational strains of the structure. The structure was finally optimized using molecular dynamics (5 ps at 298 K). Docking of the substrates and products into the active site of the model structure XrAEH was performed with the Monte Carlo method using the Docking module of the Insight II software package. The structure was further optimized using 300 minimization steps (CVFF force field) and molecular dynamics (1 ps at 298 K).

The Accelrys Discovery Studio 2.5 software package [10] was used to obtain the images of the protein globule and its complexes with the substrates.

## RESULTS AND DISCUSSION

This study included the following steps:

• multiple alignment of the XrAEH amino acid sequence with known AEH sequences to identify conserved regions (primarily the active site residues) and to select the optimal structure to be used as a reference;

• building of the three-dimensional structure of XrAEH with the homology modeling method using the reference enzyme selected at the preceding step;

• refinement of the determined XrAEH enzyme structure; and

• docking of various substrates and products of the enzymatic reaction into the model structure of XrAEH.

**Alignment of amino acid sequences of AEH from different sources**

It is known that accuracy in modeling is primarily impacted by two factors: the degree of homology between the modeled and the reference enzymes that are used as standard structures, and the resolution of the reference structure. Furthermore, even provided that homology is high, the modeling accuracy highly depends on the number and length of the gaps/insertions in the amino acid sequence alignment of the modeled and reference enzymes. The fewer the gaps/insertions, the higher the simulation accuracy will be. Therefore, in order to select the reference structure, we carried out the alignment of the amino acid sequence of the enzyme under study and two AEH sequences with known structures: from *X. citri *(XcAEH) and *A. turbidans *(ActAEH), as well as two highly homologous AEH from *X. campestris pv*. *campestris *and *X. campestris oryzae*. Note that the data on AEH from *A. pasteurianus *(which is completely identical to ActAEH in terms of the amino acid sequence) have been published; for this reason it was left out in the alignment.

**Fig. 1:**
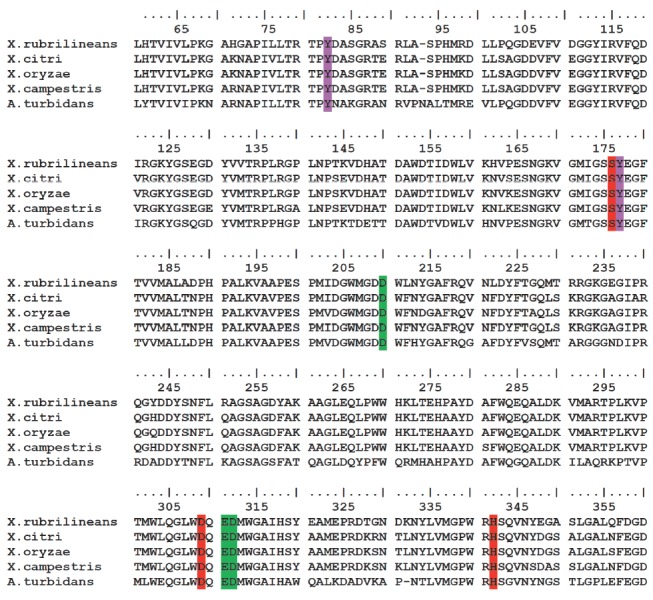
The multiple alignment of the amino acid sequences of XrAEH, AEH from *X. citri*,* X. campestris pv*.* campestris*,* X. oryzae, *and* A. turbidans *in the active site area. The catalytic triad residues, two Tyr residues from the oxyanion hole and three residues of the carboxylate cluster, are shown in red, purple, and green, respectively

The alignment results are shown in *Fig. 1*. The alignment data analysis shows that XcAEH shows the highest homology to XrAEH (84%). The homology of AEH from *X. campestris pv*. *campestris *and *X. campestris oryzae *is slightly lower (83%). The homology between XrAEH and ActAEH is much lower (62%). Moreover, *Fig. 1* shows that the alignment of the amino acid sequences of the enzyme under study and other AEH from *Xanthomonas *bacteria has no deletions or insertions, while there is one deletion and one insertion of an amino acid residue in the case of ActAEH.

Thus, based on the results of the alignment of two experimentally determined structures (ActAEH and XcAEH), the structure of the XcAEH enzyme (PDB ID: 1MPX [6]) was chosen as the reference one. In addition, the selected XcAEH 1MPX structure had a slightly higher resolution than that of the unbound ActAEH 2B9V (1.9 and 2.0 A, respectively).

**Analysis of the active site of XrAEH**

The data on the alignment of the amino acid sequences enable to determine the functionally important residues of the active site of XrAEH. Unlike penicillin G acylase (PA), which consists of two different subunits, XrAEH is a homotetramer of four identical subunits with the active site located inside each subunit. According to X-ray diffraction analysis data [4-7], the presence of three types of key amino acid residues is a characteristic feature of α-amino ester hydrolase:

1) The proton relay system to activate the catalytic serine residue. This is the typical catalytic triad of serine hydrolases; in XrAEH enzyme, it consists of Ser175, His341, and Asp308 residues (*Fig. 1*);

2) An oxyanion center consisting of two Tyr83 and Tyr176 residues in the XrAEH enzyme; it is required to stabilize the negative charge on the catalytic Ser175 residue; and

3) A carboxylate cluster consisting of three carboxyl groups of two aspartic acid residues (Asp311, Asp209) and a glutamic acid residue (Glu310). The negatively charged carboxylate cluster is involved in the binding of the positively charged amino-group of the acyl moiety of the substrate at the α-position; this binding ensures the high specificity of XrAEH to α-amino acids.

Furthermore, the Tyr223 residue is functionally important as it is involved in the binding of the phenyl moiety of the substrate due to the stacking interaction contributing to the correct orientation of the substrate in the active site of the enzyme.

**Computer modeling of the XrAEH structure**

**Fig. 2:**
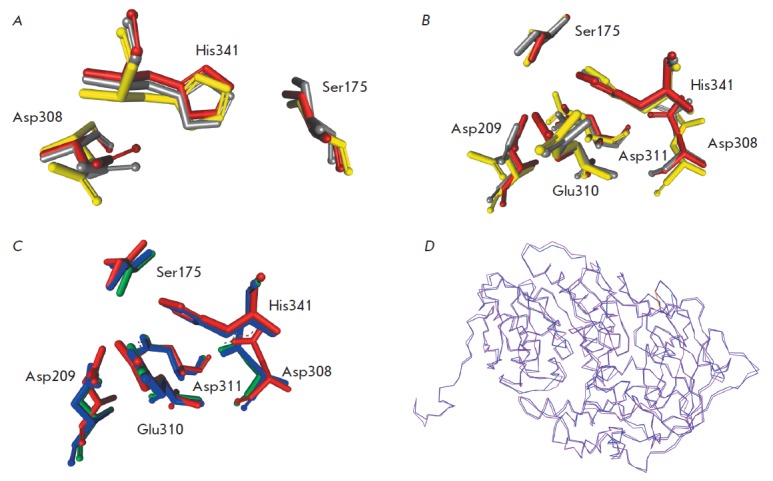
A and B – optimization of the active site structure in the model structure of XrAEH. The mutual orientation of the catalytic triad residues only and that of both the catalytic triad and carboxylate cluster residues are shown in Figs. A and B, respectively. Residues in structures with a negative net charge –3, –2, and –1 in the carboxylate cluster are shown in yellow, grey, and red, respectively. C – superimposition of the active site and carboxylate cluster residues in the model XrAEH structure (shown in red) and the experimental XcAEH and ActAEH structures (shown in green and blue, respectively). D – superimposition of C*α*-atoms of the XrAEH and XcAEH structures (shown in purple and blue, respectively). The residue numbering is given according to the XrAEH sequence

The 3D structure of XrAEH was built in two steps. First, the preliminary structure of the tetrameric enzyme XrAEH [11] was obtained using the homology modeling method with the SWISS-MODEL server. This structure was further optimized by relaxing the structure to relieve potential conformational strains using 300 steps of minimization with the Discover_3 module of the Insight II software package. An analysis of the active site structure in the model XrAEH structure obtained at this step showed that the mutual orientation of the Ser175, His341, and Asp308 residues constituting the catalytic triad is not optimal for ensuring a catalytic function (Fig. 2 A, B, residues are shown in yellow). Figure 2 demonstrates that the carboxyl group of the Asp308 residue faces away from the imidazole ring of His341. It has been suggested that this non-optimal orientation can be associated with the too-high negative charge assigned to the negatively charged carboxylate cluster consisting of carboxyl groups of the Asp209, Glu310, and Asp311 residues during the simulation. The negative charge was initially assigned to all the carboxyl groups in the residues of the carboxylate cluster of the original structure, thereby resulting in a net charge of –3. It is known that close positioning of the carboxyl groups in polymers typically prevents complete dissociation of all these groups. Therefore, we performed an additional optimization of the structure assuming that the net charge on the carboxylate cluster was equal to –2 (Fig. 2 A, B, residues are shown in gray) and –1 (Fig. 2 A, B, residues are shown in red). Figures 2A, B show that along with a decrease in the total negative charge of the carboxylate cluster the orientation of the carboxyl group of the Asp308 residue in the catalytic triad with respect to the imidazole ring of His341 becomes closer to a correct orientation. Along with this, the OH-groups of the catalytic Ser175 residue move towards the imidazole ring of His341 (Fig. 2 A). As a result, configuration of all the residues of the catalytic triad is optimal for the reaction. In addition, the negative charge of –1 at the carboxylate cluster is sufficient for the binding of the positively charged amino group of the substrate. After binding, the carboxylate cluster has no negative charge, thus suppressing the dissociation of the OH group of the catalytic residue Ser175.

Figure 2C shows the results of overlapping of the catalytic triad and carboxylate cluster residues of the optimized model of the XrAEH structure with respect to the same residues in the ActAEH and XrAEH structures determined through an X-ray diffraction analysis (PDB ID: 2B9V [5] and 1MPX [6], respectively). Figure 2C clearly shows that the spatial arrangement of the active site residues is almost identical in all three structures: the catalytic residues Ser175 and His341 and the carboxylate cluster occupy the same positions, while only a subtle deviation in the conformation of Asp308 is observed.

Figure 2D shows overlapping of the C*α*-atoms positions in the XrAEH and XcAEH structures. The figure also shows that the overall folding of the overlapping enzymes is almost identical, with the smallest deviation observed in the vicinity of the active site and the largest one observed at the periphery of the protein globule. The standard deviation of the positions of Cα- atoms in the model XrAEH structure and the reference XcAEH structure was just 0.7 A. In the case of overlapping between the XrAEH and ActAEH structures, the standard deviation was 1.1 A, as could be expected considering the lower homology between these enzymes.

**Fig. 3:**
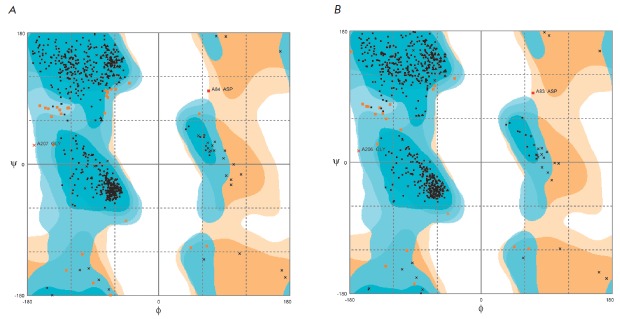
The Ramachandran plot for the model XrAEH (A) and experimental apo-XcAEH (PDB 1MPX) structures (B). The difference in the residue numbering is due to the presence of the Met residue at the N-termini in the XrAEH sequence, while the starting Met residue is not included in the amino acid sequence of XcAEH [6]

**Fig. 4:**
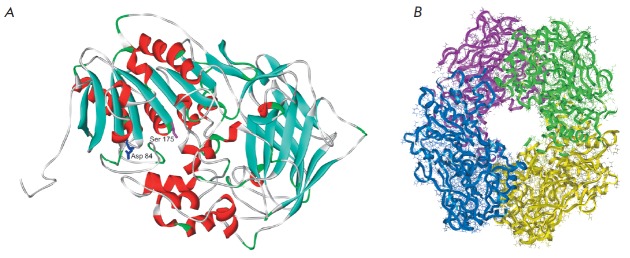
General view of one subunit (A) and the XrAEH tetramer (B)

A comparative analysis of the resulting model structure was carried out to identify residues with a nonoptimal configuration. Ramachandran maps were constructed for the model XrAEH structure and the experimental XcAEH structure (Figs. 3A, B, respectively). Figure 3 clearly shows that most residues in both structures localize in the areas of the optimal ψ and φ values. In fact, Asp84 in XrAEH and Asp83 in XcAEH are the only residues with non-optimal conformations. However, the ψ and φ values in these residues in the model and experimental structures are very close. This residue is located near the entrance to the active site in the vicinity of the bend between α-helix and β-strand (Fig. 4A). This fact means that there is a degree of strain between these subunits. The reason for such a deviation from the optimal angles is unclear. However, it should be noted that such deviations are often encountered in residues located exactly at the bends connecting secondary structure elements. For example, the same values of the ψ and φ angles are observed in the Ala198 residue in the wild-type formate dehydrogenase from bacterium *Pseudomonas sp.101* [PDB 2NAC].

Thus, these data suggest that the model structure XrAEH is reliable and has high precision; it is also in good agreement with the structure of the reference enzyme XcAEH, as well as with that of ActAEH. Figure 4 shows the structures of the monomeric and tetrameric enzyme XrAEH. This structure was further used for the docking of substrates and products into the active site of the enzyme.

**Docking of substrates and products in the active site of XrAEH**

The next step was to fit a series of substrates and products into the active site of XrAEH. The docking procedure is described in the Experimental section. The bank of three-dimensional structures provides only data on the unbound apo-enzyme of hydrolase XcAEH, which is the structurally closest homolog of our enzyme. For this reason, the structures of the XrAEH complexes resulting from docking were compared to the same or similar ActAEH structures determined experimentally.

**Fig. 5:**
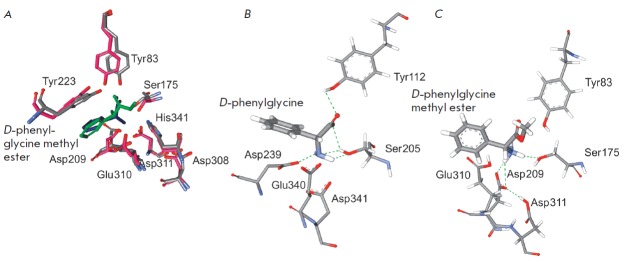
A – superimposition of the structures of the Met-DPG complex with XrAEH and the DPG complex with ActAEH. ActAEH, XrAEH, Met-DPG, and DPG are shown in pink, grey, green, and blue, respectively. The residue numbering is given according to the XrAEH sequence. B and C – interaction of active site residues with the bound ligand in the ActAEH complex with DPG and the XrAEH complex with Met-DPG

The structure of the ActAEH complex with *D*-phenylglycine (DPG) is available in the PDB (PDB ID: 2B4K [5]). However, in the case of XrAEH, the structure of its complex with *D*-phenylglycine methyl ester (Met-DPG), which is used as an acylating agent in a AEH-catalyzed synthesis of ampicillin, is of greater interest. Figure 5A shows the overlap between the obtained structure and the 2B4K structure. It can be seen that the overall folding of the structures of binary complexes is very similar; the standard deviation of C*α*-atoms for the entire protein globule is 1.1 A (note that the standard deviation for all C*α*-atoms of the protein globules of the unbound XrAEH and ActAEH enzymes was also 1.1 A). Apart from the general folding, almost complete match of the conformations of several active site residues is observed (i.e. imidazole ring of His341 residue and carboxyl group of Asp308 residue of the catalytic triad, the carboxyl groups of the Glu310 and Asp311 residues in the carboxylate center). However, the results of the overlay show noticeable differences in the conformation of other residues. Primarily, these include the hydroxyl group of the catalytic residue Ser175 and the phenolic group of the Tyr83 residue at the oxyanion center, as well as the amino group of the Met-DPG substrate. A thorough analysis of the experimental and model structures (Figs. 5B, C) with the hydrogen atoms shown provides an explanation for these differences. In the experimental 2B4K structure (Fig. 5B), there is a ActAEH complex with the reaction product. In this complex, the active site residues Ser205 and Tyr112 (Ser175 and Tyr83 in XrAEH, respectively) are positioned extremely improperly for catalysis; i.e., the hydrogen atom of the hydroxyl group of the Tyr112 phenolic ring forms a hydrogen bond with the oxygen atom of the DPG carboxyl group. As a result, the phenolic ring is fixed far away from the oxy group of the catalytic Ser205 and, therefore, cannot act as an oxyanion center in this conformation. In turn, the oxy group of the catalytic Ser205 participates in the formation of three hydrogen bonds, wherein the hydrogen atom is rotated towards the imidazole ring of the His residue due to the formation of two hydrogen bonds. The above His residue accepts this proton to produce a negatively charged oxygen atom at the Ser residue, which is required for the catalysis. In addition, the amino group of DPG is also turned away from the carboxylate center due to the formation of two hydrogen bonds with the hydroxyl group of the catalytic Ser205. As a result, only one carboxyl group of the Asp239 residue (Asp209 in XrAEH) interacts with the amino group of DPG (Fig. 5B).

**Fig. 6:**
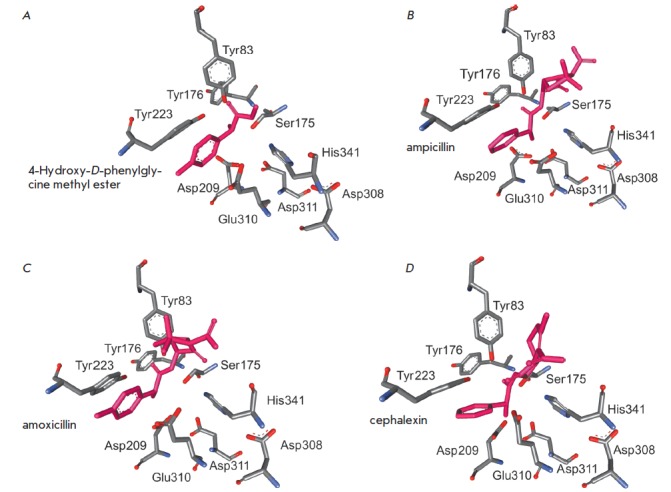
A–D –docking of 4-hydroxy-*D*-phenylglycine methyl ester, ampicillin, amoxicillin, and cephalexin in the active site of XrAEH, respectively

A totally different picture is observed in the model structure of the XrAEH complex with the Met-DPG substrate (Fig. 5C). Figure 5C clearly shows that the Fig. 5. A – superimposition of the structures of the Met-DPG complex with XrAEH and the DPG complex with ActAEH. ActAEH, XrAEH, Met-DPG, and DPG are shown in pink, grey, green, and blue, respectively. The residue numbering is given according to the XrAEH sequence. B and C – interaction of active site residues with the bound ligand in the ActAEH complex with DPG and the XrAEH complex with Met-DPG* A B C D*-phenylglycine methyl ester* D*-phenylglycine* D*-phenylglycine methyl ester phenol group of the Tyr83 residue has an optimal conformation to act as an oxyanion center; the oxygen atom of the hydroxyl group of the Ser175 catalytic residue forms only one hydrogen bond, and the hydrogen atom of this group is rotated towards the imidazole ring of the His341 residue belonging to the proton transfer system. The distance between the O*γ *atom of Ser175 and the attacked carbon atom in the substrate is just 2.9 A, and the angle of attack is 115.1°, which is close to the value of 109.5° optimal for the tetrahedral conformation. Thus, the resulting model of the XrAEH complex with Met-DPG is optimal for catalysis in terms of configuration. A somewhat different picture is observed for the XrAEH complex with 4-hydroxy-D-phenylglycine methyl ester, which is used as an acyl group donor in the synthesis of amoxicillin (Fig. 6A). The additional hydroxyl group in the aromatic ring of this substrate causes some steric hindrance when it is built into the active site of the enzyme. As a result, the angle of attack between the carbon atom of the carboxyl group and the O*γ *atom of Ser175 increases to 128.4° (Table), which is certainly worse than that in the case of Met- DPG, but still enough for the reaction to proceed efficiently.

We have also modeled the structures of the XrAEH complexes with the desired products of antibiotics synthesis reactions: ampicillin and amoxicillin (penicillin group) and cephalexin (cephalosporin group). The docking results are shown in Figs. 6B-D. According to overlay of the structures of the ampicillin and amoxicillin complexes with XrAEH, the standard deviation of C*α*-atoms for the entire protein globule is just 0.005 A; however, the conformations of the antibiotics bound to the active site are different. Identically to the case of substrates (acyl moiety donors), the distance between the O*γ *atom of the catalytic residue Ser175 of the enzyme and the carbon atom of the amide group of the product (or carboxyl carbon in the substrate) is 2.7, 3.0, and 2.9 A for ampicillin, amoxicillin, and cephalexin, respectively, but the angles differ sharply. For ampicillin, the angle is 80.9°, which is much less than the optimal value of 109.5°. For cephalexin (the angle is 73.0°), this difference is even greater. Thus, the probability that these two antibiotics are hydrolyzed in the active site of XrAEH is very low. This is not the case for amoxicillin with an attack angle of 103.2°, which is close to the optimal value. This fact means that in the case of amoxicillin, the ratio between the synthesis and hydrolysis rates (and, consequently, the yield of the target product) will be lower as compared to that of ampicillin, which is in close agreement with the experimental data [12] obtained by studying the efficacy of the recombinant enzyme in the synthesis of these antibiotics. However, note that the absolute efficacy of recombinant XrAEH in the synthesis of amoxicillin was higher than that of penicillin acylase from *E. coli*.

**Table:** The numerical results of the binding of substrates and products of the enzyme reaction in the active site of the model XrAEH structure

Embedded molecule	Distance from Oγ Ser175, Å	Angle of attack of atom Oγ Ser175, deg.
D-phenylglycine methyl ester	2.9	115.1°
Ampicillin	2.7	80.9°
4-hydroxy-D-phenylglycine methyl ester	2.9	128.4°
Amoxicillin	3.0	103.2°
Cephalexin	2.9	73.0°

Thus, we have modeled the structure of a new α-amino acid ester hydrolase from *X. rubrilineans *in the present study. In addition, the model structures of the complexes of this enzyme with a series of substrates and products have been obtained. The analysis of these structures showed good agreement with the experimental data for this enzyme, as well as for other AEHs, which is indicative of high-precision modeling. We believe that the most interesting data are the results of modeling of the structure of the XrAEH complex with amoxicillin, which is a far more efficient (and more expensive) antibacterial drug than ampicillin. For this reason, amoxicillin is used in combination with clavulanic acid, an inhibitor of β-lactamase (trade names “Augmentin”, “Clavamox” and other). As mentioned above, the penicillin acylase used today is an efficient biocatalyst for ampicillin synthesis, but it shows much lower efficiency in the synthesis of amoxicillin. Therefore, searching for and designing new biocatalysts for amoxicillin synthesis are topical tasks for the pharmaceutical industry. Availability of a model structure of the XrAEH complex with amoxicillin offers an opportunity for increasing XrAEH efficacy in the synthesis of amoxicillin using the rational design, one of the most efficient methods for protein engineering.

## Abbreviations

AEH: alpha-amino acid ester hydrolase
PA: penicillin acylase
XrAEH: alpha-amino acid ester hydrolase from Xanthomonas rubrilineans, respectively
XcAEH: alpha-amino acid ester hydrolase from Xanthomonas citri, respectively
ActAEH: alpha-amino acid ester hydrolase from Acetobacter turbidans, respectively
Met-DPG: D-phenylglycine methyl ester
DPG: D-phenylglycine
